# Physicochemical Characterization and Antioxidant Properties of Chitosan and Sodium Alginate Based Films Incorporated with Ficus Extract

**DOI:** 10.3390/polym15051215

**Published:** 2023-02-28

**Authors:** Saurabh Bhatia, Ahmed Al-Harrasi, Yasir Abbas Shah, Muhammad Jawad, Mohammed Said Al-Azri, Sana Ullah, Md Khalid Anwer, Mohammed F. Aldawsari, Esra Koca, Levent Yurdaer Aydemir

**Affiliations:** 1 Natural and Medical Sciences Research Center, University of Nizwa, P.O. Box 33, Birkat Al Mauz, Nizwa 616, Oman; 2 School of Health Science, University of Petroleum and Energy Studies, Dehradun 248007, India; 3 Center for Transdisciplinary Research, Department of Pharmacology, Saveetha Dental College and Hospital, Saveetha Institute of Medical and Technical Sciences, Saveetha University, Chennai 600077, India; 4 Department of Pharmaceutics, College of Pharmacy, Prince Sattam Bin Abdulaziz University, Al-Kharj 11942, Saudi Arabia; 5 Department of Food Engineering, Adana Alparslan Turkes Science and Technology University, Adana 01250, Turkey

**Keywords:** *Ficus racemosa*, chitosan, sodium alginate, edible films, food packaging, antioxidant activity

## Abstract

Aqueous extract of fruit obtained from *Ficus racemosa* enriched with phenolic components was used for the first time to fabricate chitosan (CS) and sodium alginate (SA)-based edible films. The edible films supplemented with Ficus fruit aqueous extract (FFE) were characterized physiochemically (using Fourier transform infrared spectroscopy (FT-IR), Texture analyser (TA), Thermogravimetric analysis (TGA), scanning electron microscopy (SEM), X-ray diffraction (XRD), and colourimeter) and biologically (using antioxidant assays). CS–SA–FFA films showed high thermal stability and high antioxidant properties. The addition of FFA into CS–SA film decreased transparency, crystallinity, tensile strength (TS), and water vapour permeability (WVP) but ameliorate moisture content (MC), elongation at break (EAB) and film thickness. The overall increase in thermal stability and antioxidant property of CS–SA–FFA films demonstrated that FFA could be alternatively used as a potent natural plant-based extract for the development of food packaging material with improved physicochemical and antioxidant properties.

## 1. Introduction

Petroleum-based films are one of the factors contributing to the dramatic rise in pollution over the past few decades, and several health problems are linked to using plastics as food packaging material. To overcome these challenges, edible films prepared from natural polysaccharides, lipids, and proteins or their combinations have gained much attention during the last few years. Polysaccharides have several potential uses in edible film preparations because of their nontoxic nature, biocompatibility, biodegradability, and processibility. Chitosan is a cationic polysaccharide that has been used in film fabrication due to its excellent film-forming properties [1,2]. However, due to its innate hydrophilicity, pure chitosan films have low water resistance. Furthermore, it is challenging to form chitosan-based edible films with desirable mechanical properties, which restricts their applicability in the fabrication of films [3]. Therefore, a variety of approaches have been suggested to address these problems and enhance the qualities of chitosan-based materials, including surface coating [4], crosslinking [5], enzyme treatment [6], and combining them with other natural polymers [7]. 

Sodium alginate is an inexpensive hydrocolloid that is safe to use, biodegradable and biocompatible, and provides a strong film structure in food packaging applications [8]. Both chitosan and sodium alginate are macromolecules safe for human consumption and can be combined to fabricate edible films with the most desirable properties [9]. Moreover, biopolymer films are promising carriers for different bioactive components, such as plant extracts, antioxidants, and antimicrobial agents. 

Extensive research has been conducted on the cluster fig tree, scientifically known as *Ficus racemosa* (Moraceae), due to its various health-promoting properties and nutritional aspects. The fruit of *Ficus racemosa* contains several pharmacologically active compounds such as glauanol, hentriacontane, -sitosterol, tiglic acid, -sitosterol, cycloartenol, cycloeuphordenol, euphol, euphorbinol, isoeuphorbol, and palmitic acid as evidenced by various studies [10]. In different studies, the fruit extract of *Ficus racemosa* has shown various biological activities such as hypoglycaemic, antioxidant, gastroprotective, and anti-filarial activity [11,12,13]. Different studies have demonstrated the safety and nontoxicity of *Ficus racemosa* fruit extract [13,14]. The incorporation of naturally occurring sources of antioxidants and antimicrobials in edible films such as plant extracts has been studied extensively. According to the findings of several studies, the incorporation of a variety of fruit extracts into edible films enhances the antioxidant activity of the films [15]. Chitosan-based edible films were prepared with the addition of *Berberis crataegina* fruit extract. Compared to other films, the film containing fruit extract showed improved thermal stability and antioxidant and antibacterial activities [16]. 

Despite its high food and medicinal potential, so far, aqueous fruit extract of *F. racemosa* has not been used in edible films to study its impact on its physiochemical characteristics. As a result, the purpose of the current study is to examine the physiochemical properties and antioxidant potential of the chitosan–sodium alginate-based edible films incorporated with *Ficus racemosa* fruit aqueous extract (FFE). 

## 2. Materials and Methods

### 2.1. Chemical Procurement

*Ficus racemosa* aqueous fruit extract (FFE) was procured from Ajmera Pharmaceutical, Indore, India. Sodium alginate (pure) and chitosan (extra pure, 90% DA) were purchased from Sisco Research Laboratories (SRL), Mumbai, India. BDH Laboratory, London, England, supplied the glycerol used as a plasticizer in films. Additionally, other required chemicals such as 2,2′-diphenyl-1-picrylhydrazyl (DPPH), butylated hydroxyl anisole, ABTS (2,2′-azinobis-(3-ethylbenzothiazoline-6-sulfonic acid)) and Trolox (6-Hydroxy-2,5,7,8-tetramethylchromane-2-carboxylic acid) were supplied by the Sigma-Aldrich (St. Louis, MO, USA). 

### 2.2. Film Preparation

The casting method was employed to form the edible films based on chitosan (CS) and sodium alginate (SA). Four types of films (FC-1–FC-4) were developed, out of which three (FC-2, FC-3, and FC-4) contained different concentrations (0.5–1.5%) of FFE, while the first (FC-1) was used as a control without FFE addition. Initially, 1% (*w*/*v*) CS solution was prepared by dissolving the polymer in 1% (*v*/*v*) acetic acid solution. Similarly, 3% (*w*/*v*) SA solution was prepared by dissolving the polymer in distilled water. Then, both solutions were mixed thoroughly in a beaker with the help of a magnetic stirrer. Following the solubilization of CS and SA, the solution was divided equally and transferred to four separate beakers (50 mL) labelled FC1–FC4. 5% glycerol was added to the film-forming solution as a plasticizer. The first beaker (FC-1) contained the CS–SA film-forming solution with 5% glycerol (*v*/*v*) and was considered the control sample in the current study. The second, third, and fourth beakers, labelled as FC-2, FC-3, and FC-4, contained 5% glycerol (*v*/*v*) film-forming solution and 0.5, 1, and 1.5% FFE, respectively. For the drying purpose, the solution was poured into Petri plates at room temperature for 48 h. After drying, the films were visually observed and then peeled out from the Petri plate’s surface for further examination. The composition of the CS–SA-based films is presented in Table 1. 

### 2.3. Thickness

A handheld digital micrometre was utilized to measure the thickness of CS–SA-fabricated films. For each prepared film sample, five random measurements were taken. The calculated mean thickness for each sample was determined in mm. 

### 2.4. Mechanical Properties of Edible Films

A standardized method (by the American Society for Testing and Materials. ASTM D882, 2010) was followed to determine the mechanical properties of the prepared film samples. Before being evaluated, the films were first conditioned for at least 40 h in a test cabinet (Nüve TK 120, Türkiye). A Universal Tester (TA. XT plus, Stable Micro Systems, UK) coupled with a 5 kg load cell was utilized to perform the experiment. The films were cut into uniform strips and inserted into the apparatus; the assessment of the mechanical properties of the films was carried out at a speed of 30 mm per minute. The mechanical properties of the films were evaluated by measuring their tensile strength (*TS*), elongation at break (*EAB*), and young’s modulus (YM). The following equations were applied to measure the mechanical assessment parameters.

(1) $$Tensile\;Strenght\;\left(TS\right)=\left(\frac{F}{A}\right)$$

*F* represents the force, and *A* shows the cross-sectional area of the film.

(2) $$Elongation\;at\;Break\;\left(EAB\right)\;\left(\%\right)=\frac{Lf-Li}{Li}\times 100$$

*Lf* presents the final length at a break, and *Li* shows the initial length of the film. 

### 2.5. Assessment of Water Solubility

The prepared film samples were examined for water solubility by following the procedure described by Kim and Song [17]. The films cut in dimensions of 3 cm by 4 cm were placed in a hot air oven at 105 °C. When films attained a constant weight, the weight at this point was noted as *W*1. The films were taken into 20 mL distilled water and placed in a shaking incubator (IKA KS3000 IC, IKA^®^-WerkeGmbH&Co. KG, Staufen, Germany) for 24 h. Then, the samples were removed from the flasks and subjected to drying in the hot air oven at 105 °C. After drying, the weight of the sample was noted as *W*2. The following equation was used to determine the water solubility of the CS–SA-based film samples.

(3) $$Water\;Solubility=\frac{W1-W2}{W1}\times 100$$

### 2.6. Moisture Content

The moisture percentage of CS–SA-based film samples was assessed by placing the film samples in an oven at 105 °C for at least three hours. The initial weight of the film samples before drying was measured as *W*1. After drying the final weight of the film samples was measured as *W*2. The moisture content (MC) was determined by the equation as follows:

(4) $$Moisture\;Content=\frac{W1-W2}{W1}\times 100$$

### 2.7. Determination of the Water Vapor Permeability

The methodology adopted by Erdem et al. [18] was employed to determine the water vapour permeability of the film samples. Glass cups with 5 cm diameter and 3 cm depth were used in the procedure. The relative humidity (RH) of the measuring systems was regulated by using water and silica gel having RH of 100% and 0%, respectively. To evaluate weight gain during the day, cups containing silica gel were covered with films that were firmly sealed and periodically weighted every hour. The WVP of the films was calculated and presented in g mm/(m^2^) (d)(kPa) by applying the below equation.

(5) $$Water\;Vapor\;Permeability=\frac{\Delta m}{\Delta t\times \Delta P\times A}\times d$$

∆*m*/∆*t* represents the weight of moisture gain per unit of time in g/d.

*A* represents the film area in m^2.^

∆*P* is the water vapour pressure difference between the two sides of the film in kPa.

*d* is the film thickness in mm.

### 2.8. Transparency

To measure the transparency of the film samples, Erdem et al.’s [18] methodology was followed by using a spectrophotometer (ONDA-vis spectrophotometer). The film samples were placed in Spectro cuvettes and transparency was measured with a spectrophotometer adjusted at 550 nm. To calculate the transparency of the films, the following formula was used, in which the *X* is the film thickness.

(6) $$Transparency=\left(\frac{A550}{X}\right)$$

### 2.9. Colour

The colour analysis of the CS–SA-based film samples was carried out by using a colourimeter CR-400 by Minolta, Tokyo, Japan. The parameters such as lightness (L*) and yellow-blue (b*) and red-green (a*) were assessed. Different positions of the film surface were used for the colour analysis. The overall colour difference, denoted by the symbol delta E, was determined by calculating the following formula:

(7) $$\Delta E={\left[{\left(\Delta L^{*}\right)}^{2}+\left(\Delta a^{*}\right){\;}^{2}+{\left(\Delta b^{*}\right)}^{2}\right]}^{1/2}$$

### 2.10. Thermogravimetric Analysis

Using a TG analyser (TA Instruments New Castle, DE 19720, USA) thermal gravimetric examination of films was performed. Composite films were scanned from room temperature to 600 °C at a heating rate of 10 °C/min under constant purging with nitrogen gas.

### 2.11. X-ray Diffraction

For analysing the XRD pattern of fabricated edible films, a Bruker D8 Discover instrument was used. The samples were examined at a 2θ diffraction angle and 40 kV voltage and a current in the range of 5–50° at a rate of 0.500 s/point, and the Scherrer constant (K) was 1.5418 Å.

### 2.12. FTIR Analysis

An FTIR Spectrometer (InfraRed Bruker Tensor 37, Ettlingen, Germany) was used to determine the elemental structure of the samples by setting them up with an attenuated total reflection (horizontal) device (45° ZnSe) [19]. For each spectrum, 32 scans in the range of 400–4000 cm^−1^ with a resolution of 4 cm^−1^ were performed. All the measurements were conducted at room temperature. 

### 2.13. SEM Analysis

The surface and cross-sectional structural features of the EFs were assessed by using SEM (JSM6510LA, Analytical SEM, Jeol, Japan) at 10 kV [19]. The films were coated with gold prior to taking images. The analysis was carried out at an acceleration voltage of 10 kV under high vacuum mode. The samples were placed on an aluminium stub covered with adhesive tapes and gold sputter-coated.

### 2.14. Antioxidant Activity

The addition of plant extracts in edible films significantly affects the antioxidant activity. The antioxidant activity of CS–SA-based film samples was evaluated using two methods, DPPH and ABTS. The methodology described by Brand-Williams et al. [20] was employed to determine the DPPH radical scavenging activity of 12.5 mg of film samples (FC-1–FC-4). A spectrophotometer (Labart LFD-10N, Italy) was used to measure the absorbance value of the film samples at 517 nm. The results obtained for the DPPH radical scavenging activity were presented as % inhibition. 

For the ABTS assay, the methodology of Re et al. [21] was used with slight modifications. In the present work, samples were measured at 734 nm after vortexing 6.25 mg of film for 30 s with 1.9 mL of 7 mmol/L ABTS radical solution produced with potassium persulfate solution (2.45 mM). The results for the ABTS radical scavenging activity were presented as % inhibition. 

### 2.15. Statistical Analysis

The statistical analysis was carried out to check the significance level between various samples. The results were presented as mean value and standard deviation. A one-way analysis of variance was conducted using statistical analysis software, followed by Duncan’s test with a 5% significant level. 

## 3. Results and Discussion 

### 3.1. Thickness 

The results for the thickness showed a slight increase with the incorporation of the FFE. FC-4 and FC-3 samples with 1.5% and 1% extract demonstrated the maximum thickness (0.05 mm), followed by FC-2 and control (Table 2). The results obtained are in agreement with the findings of Kumar et al. [22], in which the chitosan-based edible films exhibited similar behaviour when incorporated with pomegranate peel extract.

### 3.2. Mechanical Properties

A composite film meant for food packaging must possess appropriate mechanical properties including resistance to common external forces during handling, shipping, and storage. The CS–SA-based edible film samples were examined for their mechanical properties and the obtained results are expressed in Table 2. The tensile strength (TS) of the control or FC-1 sample without FFE addition was relatively higher than FC-2, FC-3, and FC-4 samples. The tensile strength of the FC-4 samples with 1.5% FFE was lower than other samples. The decrease in the TS of the films with increasing the extract concentration could be ascribed to the weak molecular interaction between the film-forming components with the incorporation of FFE. 

However, the EAB value for different CS–SA-based film samples increased from 16.21–21.47%. The control or FC-1 sample without FFE addition exhibited the lowest values (16.21%) compared to other samples comprising FFE. The EAB value of the FC-4 sample (21.47%) was higher than other samples; this could be due to the increase in molecular mobility by adding extract. The results of our study are corroborated with the findings of Nemazifard et al. [23] where a decrease in TS and an increase in EAB among cellulose films incorporated with pomegranate seed extract is reported. Zhang et al. [24] also reported similar behaviour of the mechanical characteristics of CS films with the addition of banana peel extract. However, the mechanical properties of the films also depend upon different factors such as film constituents, types of polymers, processing technique, drying conditions, moisture, etc. 

### 3.3. Moisture Content and Water Solubility

For food applications, edible films should be preferably water-resistant. However, natural polymer-based films usually have poor water resistance (Al-Harrasi et al., 2022). In the current study, an increase in the moisture content among FC-1–FC-4 samples was observed due to the incorporation of the FFE (Table 2). The maximum (34.82%) and minimum (24.16%) moisture content was found in FC-4 and control, respectively. This could be due to the hydrophilic nature of the polymers as well as the extract added. Recent studies have shown that adding plant extracts in biopolymer-based films enhances their water moisture and solubility [25]. In a previous study, conducted by Augusto et al. [26], the researchers found that the incorporation of *Codium tomentosum* seaweed extract led to an increase in the amount of moisture present in edible films based on chitosan and alginate. 

The water solubility of the CS–SA-based edible film samples showed 100% values for all the film samples. This behaviour could be ascribed to the hydrophilic nature of the film-forming components, including chitosan and sodium alginate, as well as FFE. 

### 3.4. Water Vapor Permeability

The composition of the films generally determines the water vapour permeability because the hydrophobic compounds present in the film prevent the transfer of moisture from the surroundings to the packaged food. Ideally, food packaging materials should have lower WVP values, thereby reducing food spoilage [22]. The results for the WVP of tested film samples have been presented in Table 2. The WVP value of the control film sample (0.356) was greater than the samples with the added extract. The lowest water permeability was observed in FC-4 (0.333), followed by FC-3 (0.337) and FC-2 (0.348). 

A decline in the WVP was observed with an increase in the concentration of the FFE. This behaviour could be due to good intermolecular interaction between the film-forming components with the addition of FFE. The findings of our study are in line with Talón et al. [27], who reported a decline in the WVP of the chitosan–starch-based films when incorporated with thyme extract. Furthermore, Yong et al. [28] presented parallel results when chitosan-based films were incorporated with purple and black eggplant extracts. 

### 3.5. Transparency

An essential physical characteristic of edible films is transparency, which refers to the lack of visibility or resistance to light transmission. The results of the transparency of the CS–SA-based edible films are presented in Table 3. A reduction in the transparency of the films was observed by increasing the concentration of the FFE. The maximum transparency was observed in the control (FC-1) sample (80.45%). The lowest transparency was shown by FC-4 (46.427%) loaded with the highest concentration (1.5%) of FFE. This could be due to the scattering of light with colour components and phenolic compounds in the extract. Qin et al. [29] also reported similar results in which pomegranate peel extract was added to the chitosan-based films and the transparency decreased.

### 3.6. Colour Analysis

The apparent colour of the film has a significant impact on how packed food appears, and the colour is a crucial determinant of packaging acceptance by the end consumers. According to the literature, adding plant extracts changes the original colour of the films, but the change is dependent on the source and quantity of the extracts [30]. The colour parameters of the FC-4 sample with a maximum concentration (1.5%) of FFE shifted from transparent to blue-yellow (b*). The control sample (FC-1) was more evident as compared to samples with extract added (Table 3). Adding the FFE slightly decreased the lightness (L*) of the film samples. The current study results demonstrate that adding FFE significantly affected the colour parameters of the film samples.

Zhang et al. [24] also reported an increase in the yellow colour of the chitosan-based edible films with the incorporation of banana peel extract. Moreover, in the results of our study, the difference in the colour parameters could be ascribed to the different concentrations of the FFE.

### 3.7. TGA

**Figure 1:**
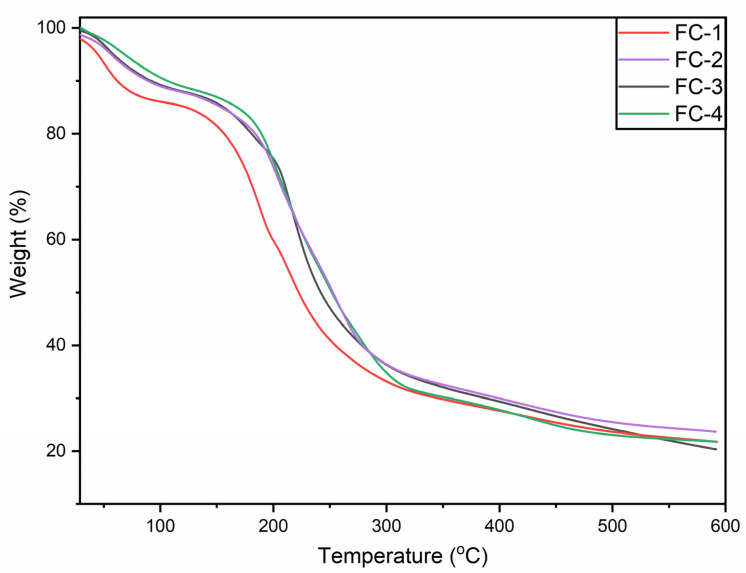
Thermogravimetric analysis of CS–SA edible film samples: control or FC-1, CS + SA; FC-2, CS + SA + FFE (0.5%); FC-3, CS + SA + FFE (1%); and FC-4, CS + SA + FFE (1.5%).

The thermogravimetric analysis of the film samples was performed to investigate the impact of FFE on the thermal stability of the CS–SA film samples. The examined film samples exhibited similar cycles of weight loss in the TG curve (Figure 1). In the temperature range of 35 to 130 ° C, the occurrence of first thermal degradation could be associated with water evaporation [2]. A weight loss of approximately 10% was observed during the first stage in all the samples, excluding the control (FC-1), which had a relatively dramatic weight loss compared to the other samples. This weight loss could be due to the thermal degradation of polymers having low intermolecular interaction as the addition of FFE would have improved the interaction among the polymers [31]. 

The second weight loss phase was observed for all samples between 150 and 400 °C. It could be due to the thermal degradation of film-forming components, including sodium alginate, chitosan, glycerol, and FFE. According to reports, glycerol-rich material, chitosan, and sodium alginate break down between 150–260 °C, 240–360 °C, and 229–243 °C, respectively [32,33,34]. The FC-4 sample with 1.5% extract showed good thermal stability compared to the control, FC-2, and FC-3. The results obtained from the thermogravimetric analysis demonstrate that FFE enhanced the intermolecular interaction between chitosan and sodium alginate; hence, thermal stability of the polysaccharide chains improved. 

### 3.8. XRD

**Figure 2:**
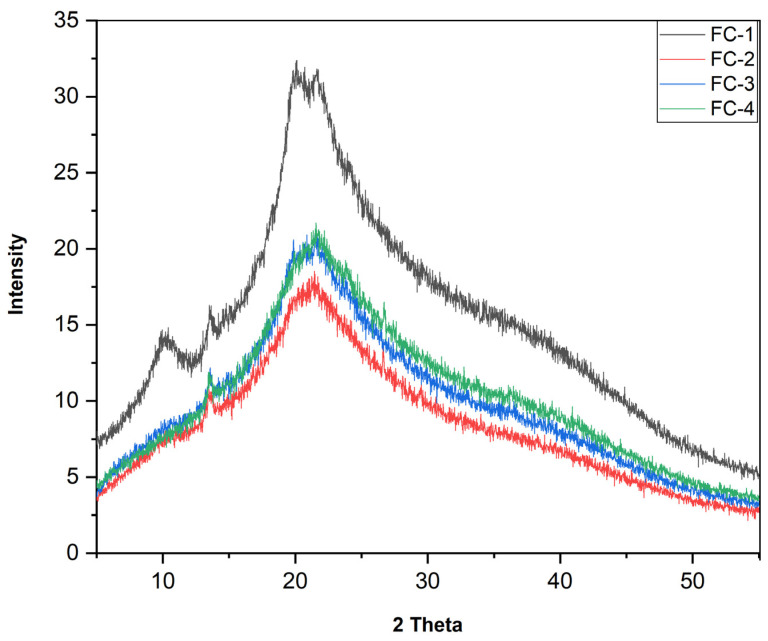
XRD of CS–SA edible film samples, control or FC-1, CS + SA; FC-2, CS + SA + FFE (0.5%); FC-3, CS + SA + FFE (1%); and FC-4 CS + SA + FFE (1.5%).

The XRD patterns of examined film samples, including FC-1, CS + SA; FC-2, CS + SA + FC (0.5%); FC-3, CS + SA + FC (1%); and FC-4, CS + SA + FC (1.5%) is shown in Figure 2. The control (FC-1) sample showed a broader peak at 20° of 2θ and two small peaks, one peak at 10.21° of 2θ, and a small sharp peak at 13.5. The characteristic peak at 10.21° of 2θ disappeared with the incorporation of FFE in film samples. A broader peak was observed for FC-2, FC-3, and FC-4 samples at 20° of 2θ as well as one small sharp peak being observed for all the samples at 13.7° of 2θ. In our previous work, all the edible film samples of chitosan and sodium alginate exhibited a characteristic peak at 14° of 2θ [19].

The results of the current study show the partial crystalline nature of CS–SA-based film samples incorporated with FFE. The percentage of crystallinity of the FC-1, FC-2, FC-3, and FC-4 film samples was 18.3%, 14.5%, 15.1%, and 15%, respectively. The addition of FFE slightly decreased the crystallinity of CS–SA-based edible films. The decrease in peak intensity could be attributed to the intermolecular interaction between CS–SA and FFE, which reduces molecule mobility and thus prevents crystallization. The difference in the concentrations of FFE added to the films could be the reason for the different peak intensities observed. The alterations in the peak intensities as a result of alteration in the concentration of film-forming components could be correlated with the mechanical characteristics of the edible films [19]. 

### 3.9. FTIR Analysis

**Figure 3:**
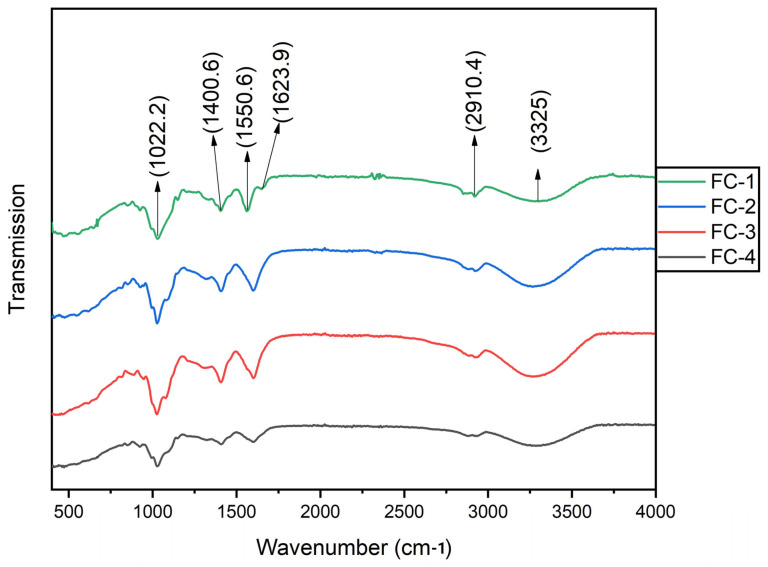
FTIR of CS–SA edible film samples, control or FC-1, CS + SA; FC-2, CS + SA + FFE (0.5%); FC-3, CS + SA + FFE (1%); and FC-4, CS + SA + FFE (1.5%).

The interactions between the functional groups of film-forming components including chitosan, sodium alginate, and the FFE were examined. The FTIR spectra of the examined samples is demonstrated in Figure 3, emphasizing the molecular interaction of CS, SA, and FFE. Most of the samples’ FTIR patterns were approximately similar, with a slight difference in the transmission intensity. The peak intensity alteration may indicate the change in extract concentration added during film formation. 

The characteristic peaks noticed at 3325 cm^−1^ represent the stretching of the N–H group while the peaks at 2910.4 cm^−1^ indicate C–H stretching. The peaks observed at 1623.9 cm^−1^ and 1550.6 cm^−1^ specify the stretching of the C=C group and N–O group, respectively. Moreover, the O–H bending of carboxylic and stretching of the C–O group can be observed at 1400.6 cm^−1^ and 1022.2 cm^−1^, respectively (Figure 3). A previous study reported that chitosan and sodium alginate-based films exhibited similar patterns during the FTIR analysis [31]. Previous studies described that peaks at 1632 and 1550.6 cm^−1^ may indicate the presence of chitosan [35,36]. Studies reported that the presence of sodium alginate in the film sample could be attributed to the characteristic peaks at 2910.4 cm^−1^ and 1400.6 cm^−1^ [19,37]. 

In a recent study, the FTIR analysis of the *F. racemosa* fruit extract exhibited the stretching of the N–H or amine, stretching of the N–O group, and O–H blending of the carboxylic [38]. The characteristics peak at 3325, 1400.6, and 1550.6 cm^−1^ could be ascribed to the presence of FFE in the edible films and its interaction with film-forming polymers. FTIR analyses showed the interactions between chitosan, sodium alginate, and FFE from the corresponding peak positions. 

### 3.10. Scanning Electron Microscopy

**Figure 4:**
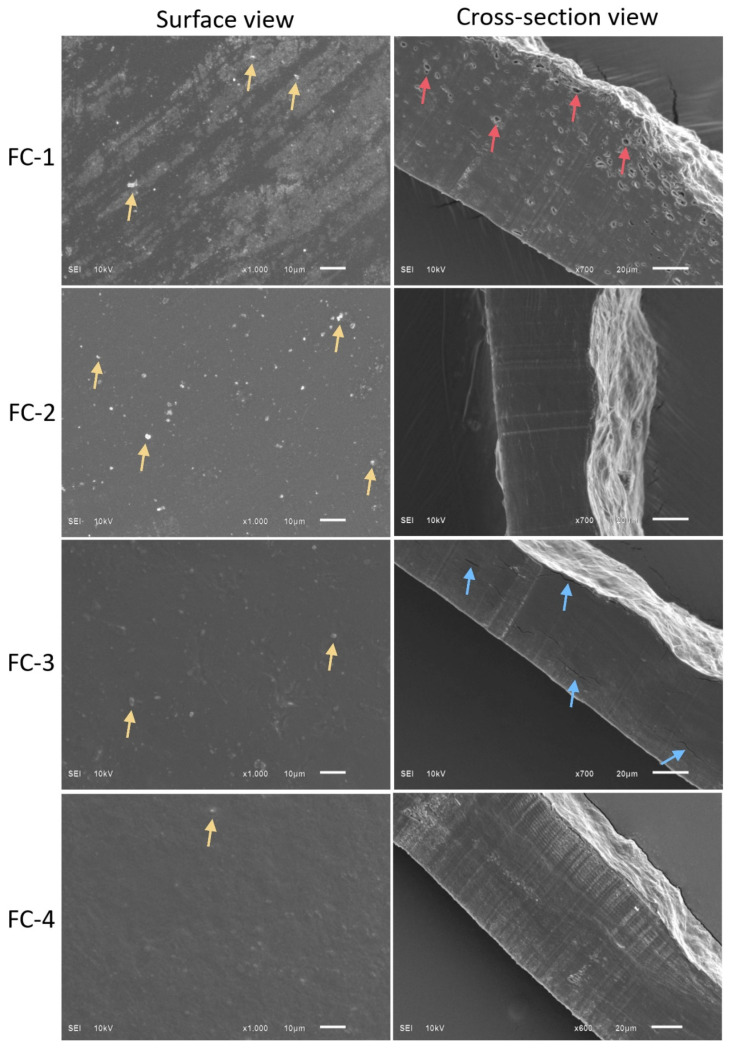
SEM of the CS–SA composite films: control or FC-1, CS + SA; FC-2, CS + SA + FFE (0.5%); FC-3, CS + SA + FFE (1%); and FC-4, CS + SA + FFE (1.5%). The colour of the arrows presents different properties; yellow indicates particles, red indicates pores, and blue indicates cracks.

The prepared CS–SA-based edible films were examined for their morphological structures. Figure 4 shows the morphological characteristics of film samples, including FC-1, CS + SA; FC-2, CS + SA + FC (0.5%); FC-3, CS + SA + FC (1%); and FC-4, CS + SA + FC (1.5%). The effect of FFE on the characteristics of CS–SA-based films was investigated by observing these samples’ microstructural properties. The structural morphology of the CS (control) FC-1 sample showed roughness, pores, and the presence of tiny particles on the surface (Figure 4). These structural discontinuities in the FC-1 sample align with the findings of Li et al. [31] where chitosan and sodium-alginate-based films showed a rough surface, indicating reduced components homogeneity in the film matrix. Previous studies have demonstrated that pores and tiny particles on the surface significantly affect the mechanical and barrier characteristics of films [39]. 

SEM characteristics of the FC-2 sample with 0.5% FFE showed less roughness and pores than the control. Still, more tiny particles on the surface and bulge structure were observed compared to FC-3 and FC-4 samples. This could be the effect of FFE at 0.5% that interfered with the plasticizing effect of Gly and the film-forming capacity of chitosan [2]. The FC-3 sample with 1% FFE showed less roughness and particles on the surface when compared with the control and FC-1 samples (Figure 4). However, the surface of the FC-3 sample was uneven with cracks compared to the control, FC-2, and FC-4 samples. The processing conditions, such as temperature conditions and film drying, could cause cracks in the film sample [40].

The SEM characteristics of the FC-4 sample with 1.5% FFE showed good morphological characteristics with a uniform structure without any pores, cracks, and fewer particles on the surface as compared to control, FC-2, and FC-3 samples (Figure 4). These morphological changes could be due to the presence of phenolic components acting as crosslinkers in the FFE [41]. It was assumed from the current analysis that increased FFE concentration possibly increased the crosslinking between chitosan and sodium alginate and thus resulted in more uniform films. 

### 3.11. Antioxidant Properties

**Figure 5:**
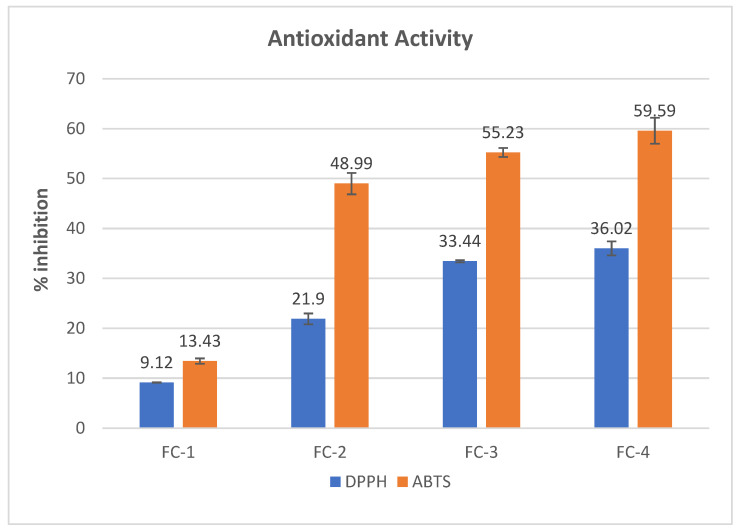
Antioxidant properties of the CS–SA-based edible films; control or FC-1, CS + SA; FC-2, CS + SA + FFE (0.5%); FC-3, CS + SA + FFE (1%); and FC-4 CS + SA + FFE (1.5%).

One of the main problems impacting food quality is oxidation-induced deterioration, and this can be minimized with the addition of natural antioxidants to food packaging materials such as edible films [42]. In the current study, FFE was incorporated into chitosan and sodium alginate-based edible films owing to its potential antioxidant activity as demonstrated by various studies [11,43]. Figure 5 shows the free radical scavenging activity of FC-1–4 samples using DPPH and ABTS radical scavenging assays. Results obtained from the DPPH radical scavenging assay showed that the FC-1 sample/control without FFE showed less antioxidant activity than FC-2, FC-3, and FC-4 samples. An increase in the antioxidant value could be attributed to different phenolic compounds present in the fruit of *F. racemosa*, as evidenced by various studies [10]. The highest % inhibition was found in the FC-4 sample with 1.5% FFE, followed by FC-3 with 1% FFE and FC-2 with 0.5% FFE. 

Similar to DPPH radical scavenging, the control/FC-1 sample without FFE exhibited less antioxidant activity, as determined by ABTS radical scavenging activity. The FC-4 sample with 1.5% FFE exhibited the highest % inhibition than control, FC-2, and FC-3. The antioxidant potential of *F. racemosa* fruit extract was evaluated by Hasan et al. [44] using the ABTS method, and the findings revealed that *F. racemosa* fruit extract contains several potent phytochemicals that present significant antioxidant activity. These findings are in alignment with work where an increase in the antioxidant activity of the chitosan films was observed when incorporated with purple and black eggplant extracts [28]. 

## 4. Conclusions

The present study reported the development, characterization and biological assessment of CS–SA films loaded with FFE. For the first time, FFE extract was used for edible film production. It was found that the incorporation of extract into the films improved the overall thermal stability and antioxidant activity. Additionally, the incorporation of plant extract into CS–SA films improved the overall elasticity and decreased the WVP and crystallinity of the films. Thus, based on the present findings, FC-4 was more suitable for food packaging. However, further research is required to improve its tensile strength and transparency without impacting its physiochemical properties.

## Figures and Tables

**Table 1:** Chemical composition of the CS–SA-based film-forming solution.

Sample Codes	The Composition of the Film-Forming Components
FC-1	CS (1%) + SA (3%) + Gly (5%)
FC-2	CS (1%) + SA (3%) + Gly (5%) + FC (0.5%)
FC-3	CS (1%) + SA (3%) + Gly (5%) + FC (1%)
FC-4	CS (1%) + SA (3%) + Gly (5%) + FC (1.5%)

Codes: CS: chitosan; SA: sodium alginate; GLY: glycerol; FC: Ficus extract.

**Table 2:** Tensile strength, elongation at break, thickness, moisture content, and water vapor permeability of the CS–SA-based edible films.

Sample Codes	TS (Mpa)	EAB (%)	Film Thickness (mm)	Moisture Content (%)	WVP (×10^−12^ g·cm/cm^2^·s·Pa)
FC-1	2.136 ± 0.150 ^a^	16.212 ± 0.373 ^a^	0.040 ± 0.007 ^a^	24.16 ± 1.07 ^ab^	0.356 ± 0.006 ^a^
FC-2	1.388 ± 0.109 ^b^	17.541 ± 0.104 ^b^	0.040 ± 0.010 ^ab^	26.66 ± 0.75 ^b^	0.348 ± 0.001 ^b^
FC-3	1.170 ± 0.046 ^c^	19.176 ± 0.581 ^c^	0.054 ± 0.009 ^b^	30.87 ± 0.46 ^c^	0.337 ± 0.009 ^c^
FC-4	1.046 ± 0.019 ^d^	21.475 ± 0.659 ^d^	0.053 ± 0.005 ^b^	34.82 ± 0.97 ^d^	0.333 ± 0.005 ^d^

Means carrying the same letters are significantly identical.

**Table 3:** Colour parameters and transparency of the CS–SA-based edible films.

Sample	*L*	a*	b*	Δ*E*	Transparency %
FC-1	96.43 ± 0.15 ^a^	−0.15 ± 0.02 ^a^	1.74 ± 0.07 ^a^	0.94 ± 0.010 ^a^	80.45 ± 0.65 ^a^
FC-2	95.63 ± 0.22 ^a^	−0.15 ± 0.02 ^a^	2.58 ± 0.22 ^b^	1.74 ± 0.17 ^b^	75.25 ± 0.12 ^b^
FC-3	95.10 ± 0.11 ^a^	−0.09 ± 0.00 ^b^	2.77 ± 0.25 ^b^	2.09 ± 0.27 ^c^	67.69 ± 0.33 ^c^
FC-4	94.94 ± 0.09 ^a^	−0.04 ± 0.01 ^c^	3.06 ± 0.07 ^c^	2.42 ± 0.09 ^d^	46.42 ± 2.04 ^d^

Means carrying the same letters are significantly identical.

## Data Availability

Not applicable.
